# Action-space coding in social contexts

**DOI:** 10.1038/srep22673

**Published:** 2016-03-04

**Authors:** Francesca Ciardo, Luisa Lugli, Roberto Nicoletti, Sandro Rubichi, Cristina Iani

**Affiliations:** 1 Department of Communication and Economics, University of Modena and Reggio Emilia, Viale Allegri, 9, 42121 Reggio Emilia, Italy; 2 Department of Philosophy and Communication, University of Bologna, via A. Gardino 23, 40122, Bologna, Italy

## Abstract

In two behavioural experiments we tested whether performing a spatial task along with another agent changes space representation by rendering some reference frames more/less salient than others. To this end, we used a Simon task in which stimuli were presented in four horizontal locations thus allowing for spatial coding according to multiple frames of reference. In Experiment 1 participants performed a go/no-go Simon task along another agent, each being in charge of one response. In Experiment 2 they performed a two-choice Simon task along another agent, each being in charge of two responses. Results showed that when participants were in charge of only one response, stimulus position was coded only with reference to the centre of the screen hence suggesting that the co-actor’s response, or the position of the co-actor, was represented and used as a reference for spatial coding. Differently, when participants were in charge of two responses, no effect of the social context emerged and spatial coding relied on multiple frames of reference, similarly to when the Simon task is performed individually. These findings provide insights on the influence played by the interaction between the social context (i.e. the presence of others) and task features on individual performance.

The results of an increasing number of studies indicate that acting in a social context affects individual performance in various ways. Most of these results derive from the use of controlled laboratory tasks in which pairs of participants are required to alternate in responding to relevant features of visual or auditory stimuli. The striking result is that each participant seems to consider not only the part of the task that is under his/her responsibility, but also the part that is under the other participant’s responsibility, even when there is no need to do so (see^1^,^2^,^3^ for reviews).

A typical task used to investigate how acting in a social context affects performance is the Simon task^4^,^5^ (see^6^ for a review). In the individual two-choice version of the task, single participants are required to respond to a nonspatial feature (e.g. colour or shape) of a stimulus presented on a computer screen to the left or right of fixation with a right or left key-press, ignoring its location. Even though stimulus location is completely irrelevant for performing the task, responses are faster and more accurate when stimulus and response position correspond (corresponding trials) compared to when they do not correspond (noncorresponding trials). The difference in performance between noncorresponding and corresponding responses, known as Simon effect, is attributed to the automatic pre-activation of the response that spatially corresponds to stimulus location^7^,^8^. In corresponding trials, this automatically activated response is the same as the one indicated by the relevant stimulus feature; therefore no competition between response codes arises. In noncorresponding trials, instead, the automatically activated response and the response activated on the basis of the relevant stimulus feature are different and the incorrect response needs to be aborted thus causing a slowing of response time and an increased number of errors.

Sebanz *et al*.^9^ showed that, while the Simon effect was absent when a single individual performed half of the task (see also^10^) responding to only one stimulus colour (i.e. individual go/no-go Simon task), the effect emerged when the task was performed jointly by two individuals, each responding to only one stimulus colour (joint go/no-go Simon task). Such a finding has been replicated in many experimental situations (e.g.^11^,^12^,^13^,^14^,^15^) and has been interpreted as an indication that when people perform together complementary parts of a task, they tend to represent the whole task and integrate their own and others’ actions in a shared representation (co-representation). In the absence of such a co-representation, no conflict between alternative responses would arise since only one response is required, as occurs in the individual go/no-go version of the task (but see also^16^,^17^,^18^,^19^ for a different account).

Such an interpretation speaks in favour of a large overlap between the cognitive mechanisms involved in the individual two-choice and in the joint versions of the Simon task. However, Guagnano and colleagues^20^ showed that if a co-actor is sufficiently close, he/she provides a spatial reference point for coding the location of one’s own action. Thus, instead of representing the specifics of the other’s task or actions, co-actors might simply use the other as a spatial reference. However, it is still unclear how exactly such a spatial reference is established. Accordingly, Welsh^21^ reported that the Simon effect evident in joint settings (from now on, Joint Simon effect or JSE) when participants performed the task with their hand crossed was similar to the effect evident when they performed the task with their hand uncrossed, suggesting that spatial coding in the JSE is flexibly based on the position of one’s body relative to the other’s body, or on the position of one’s hand relative to the other’s hand.

To note, it has been proposed that the JSE might rely not only on the stimulus-response (S-R) spatial correspondence (as is known to be crucial for the standard Simon effect to emerge), but also on the correspondence between stimulus and responding agents (e.g.^22^). According to this view, when the Simon task is shared between two individuals, spatial information may be used as an indication of whose turn it is. This would mean that a stimulus appearing on the left does not lead to the automatic activation of the left response, as in the individual version of the task, but rather is perceived as a stimulus signalling that the person sitting on the left is in charge of responding. This idea has been further elaborated by Wenke *et al*.^23^ who proposed that joint correspondence effects are not the result of the representation of the co-agent’s task but rather are due to the representation of when it is the other person’s turn (agent co-representation).

Importantly, when the S-R setting comprises left and right elements, left and right values can be specified along two dimensions, one absolute and the other relative. In the case of the absolute dimension, the stimulus can be either on the left or on the right side in relation to an egocentric reference axis, such as the body midline, the head midline, or the vertical retinal meridian. In the case of the relative dimension, the stimulus can be either on the left or on the right position in relation to an external reference point, such as another stimulus. As regards the individual two-choice Simon effect, it has been shown that stimulus location can be coded according to multiple frames of reference. For instance, Lamberts, Tavernier, and d’Ydewalle^24^ showed that when the stimulus could appear in one of eight possible horizontal locations, four on each half of the screen, a Simon effect emerged with respect to hemispace, hemifield and relative position. Hence, the spatial relationships between stimuli and responses, defined by each of the three reference frames, independently influenced performance. Roswarski and Proctor^25^ showed that the spatial coding with respect to multiple frames of reference occurs even when no cues are provided to specify the hemispace in which the target stimulus appears. However, when instructions explicitly indicate the relevant frame of reference, stimulus location is coded solely with respect to that frame (see also^26^).

So far studies employing the Simon task to investigate joint action have used simple displays in which a visual stimulus is presented on the left or on the right side of the screen, thus it can be univocally coded as left or right. To the best of our knowledge, there is no study using more complex displays in which stimuli could be coded according to multiple frames of reference. Such a situation would allow assessing whether the presence of another agent changes space representation by rendering some reference frames more/less salient than others and consequently affecting performance. This issue is particularly relevant especially considering the numerous real-life settings that may require agents to react to stimuli in the presence of others with different levels of complementarity required by the tasks. An example may be represented by the aircraft cockpit in which pilot and co-pilot have each a set of displays in front of them (i.e. the primary flight display and the navigational display) but have also shared displays presented in the middle of the front panel and are required to perform different tasks by using all these displays or to alternate in performing a task (i.e. flying the aircraft). One open question is whether and to what extent individual action-space coding is affected by the presence of an agent sitting in close proximity.

Given the above considerations, in the present study we aimed at investigating whether, in a joint action context, stimuli and responses can be coded according to multiple frames of reference. To this aim, we manipulated stimulus position in a joint Simon paradigm using the same display developed by Roswarski and Proctor^25^ (Experiment 3), in which stimuli could appear in one of four possible locations. As specified by Roswarski and Proctor^25^, this display reproduces a real-life setting. Indeed, it reproduces the layout of the cockpit environment of some fighter aircrafts in which pilots have a large panel directly in front of them and two smaller panels, one to their left and one to their right side. In this display a left and right division is created based on the midline of the front panel, and two additional left and right divisions are created where the front and left panels intersect and where the front and right panels intersect^27^. Roswarski and Proctor^25^ showed that, in the individual two-choice Simon task, this layout allows for spatial coding based on multiple frames of reference: one frame is related to the stimulus absolute position in space (i.e. left or right hemispace), while the other is related to the stimulus relative position within each hemispace (i.e. left or right relative position).

Experiment 1 was aimed at assessing whether S-R spatial coding occurs according to multiple frames of reference when individuals perform a joint Simon task, that is when each agent has one response key at his/her disposal and thus response position could be coded as right or left according to the shared frame of reference. To this end, participants sat on the left or on the right of the centre of the screen and were asked to perform a go/no-go Simon task responding to the colour of one of two stimuli. They were assigned to one of two conditions: in the Individual condition, each participant performed the task alone, while in the Joint condition two participants performed the task together. Under both conditions, each participant was in charge of only one response. According to Sebanz and colleagues (e.g.^9^,^14^,^15^,^28^) the JSE can be interpreted as an indication that when people perform together complementary parts of a task, they tend to represent the whole task and integrate their own and others’ actions in a shared representation. In the absence of such representation, no conflict between alternative responses would arise, as occurs in the individual go/no-go version of the task. We hypothesized that the Simon effect should not emerge when participants perform the go/no-go Simon task alone, since no left and right spatial coding of the response occurs. In contrast, we predicted that a left and right spatial coding should occur for the joint go/no-go task. Specifically, if the task representation guiding joint task performance is similar to the representations guiding individual performance^9^,^14^,^15^,^28^, then the left and right spatial coding should occur for multiple frames of reference as reported in the individual Simon task^24^,^25^,^26^. Thus, in the Individual condition no Simon effect should emerge either with respect to stimulus relative position or with respect to stimulus absolute position in space (i.e. the left or right hemispace), since no spatial coding of the response is required. In the Joint condition, in line with Roswarski and Proctor^25^, a JSE should be evident both for the absolute stimulus position and for its relative position. This result will indicate that for the JSE the S-R spatial coding relies on multiple frames of reference.

Experiment 2 was aimed at assessing whether spatial coding takes place according to the relevant frame of reference only, even if each agent has two response keys at his/her disposal, and thus response position could be coded as right or left according to the body midline. To this end, participants were asked to perform a complementary task in which each participant performed a two-choice Simon task. As in Experiment 1, two conditions were included: in the Individual condition, each participant performed the task alone; while in the Joint condition paired participants performed the task together. Differently from Experiment 1, four stimuli and four response keys were used and each participant responded to two stimuli by pressing two of the four keys. We hypothesized that since the response position could be coded as right or left with reference to the body midline, according to Guagnano *et al*.^20^, the co-actor does not provides a spatial reference point for coding response location, thus no difference in the Simon effect should occur across the Individual and Joint conditions. As regards spatial coding at the basis of the Simon effect, we predicted that spatial coding should rely both on stimulus’ absolute position (i.e. left or right hemispace), and on its relative position within each hemispace^24^,^25^.

## Results

### Experiment 1

Errors were 0.18% and 0.8% of the total trials for the Individual and Joint conditions, respectively, and were not further analysed. Correct reaction times (RTs) were submitted to a repeated-measures analysis of variance (ANOVA) with Hemispace (left vs. right), and Relative stimulus position (left vs. right) as within-participant factors, and Response position (left vs. right) and Condition (Individual vs. Joint) as between-participants factors. The results are summarized in Table 1. Following significant interactions, post-hoc comparisons were performed using the Newman-Keuls test.

**Figure 1:**
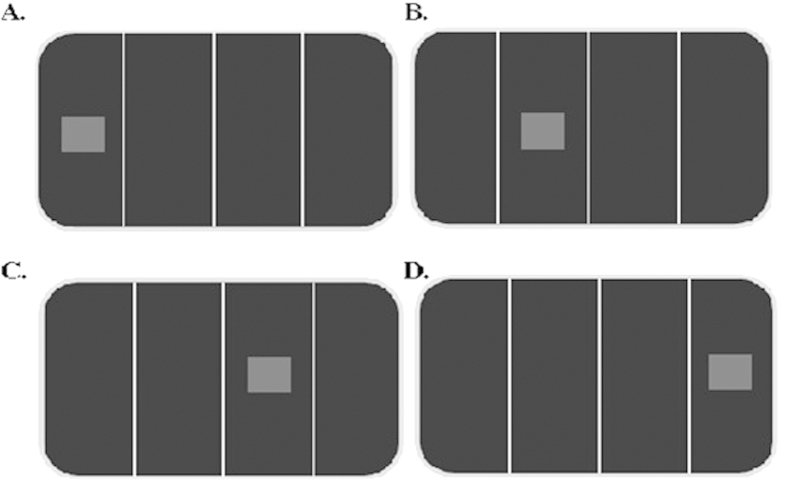
Schematic representation of the four locations in which the stimulus could appear, resulting for the manipulation of hemispace (left and right) and relative position (left and right). Left hemispace refers to panels A and B; whereas right hemispace refers to panels C and D. Left relative position refers to panels A and C; whereas right relative position refers to panels B and D.

The analysis revealed no significant main effects, all *p*s > 0.11. There was a significant interaction between Hemispace and Relative stimulus position, *F* (1, 28) = 21.31, *p* < 0.001, *η*_*p*_^2^ = 0.43. Post-hoc comparisons showed that, within each hemispace, responses to the outer stimulus position (i.e. the left stimulus position in the left hemispace and the right stimulus position in the right hemispace; Fig. 1, panels A and D) were slower (350 and 347 ms for the left and the right hemispace, respectively) than responses to the inner stimulus position (i.e. the right stimulus position in the left hemispace and the left stimulus position in the right hemispace; Fig. 1, panels B and C; 340 and 336 ms for the left and the right hemispaces, respectively), all *p*s < 0.01. No difference was evident between the two inner positions and between the two outer positions (*p*s > 0.32).

More relevant for the purpose of the present study are the interactions involving response position because they are indicative of a Simon effect. More precisely, an interaction between response position and hemispace would be indicative of a Simon effect with respect to the stimulus absolute position (i.e. hemispace), while an interaction between response position and relative position would be indicative of a Simon effect with respect to the stimulus relative position (i.e. left and right position within each hemispace). For sake of clarity, it should be noted that, with reference to hemispace, a left-located response was corresponding for stimuli appearing to the left of fixation (Fig. 1, panels A and B), and noncorresponding for stimuli appearing to the right of fixation (Fig. 1, panels C and D; vice versa for the right-located response). With reference to the relative position frame, a left-located response was corresponding for stimuli appearing in the left-most position of each hemispace (Fig. 1, panels A and C), and noncorresponding for stimuli appearing in the right-most position of each hemispace (Fig. 1, panels B and D, vice versa for the right-located response).

Crucially, both the two-way interaction between Hemispace and Response position, *F*(1, 28) = 9.87, *p* = 0.004, *η*_*p*_^*2*^ = 0.26, and the three-way interaction between Hemispace, Response position, and Condition, *F*(1, 28) = 10.85, *p* = 0.003, *η*_*p*_^*2*^ = 0.28, were significant. To further assess the three-way interaction, we ran separate analyses by Condition. These analyses showed that the two-way interaction between Hemispace and Response position did not reach significance for the Individual condition, *F* < 1, while it was significant for the Joint condition, *F*(1, 14) = 17.71, *p* = 0.001, *η*_*p*_^*2*^ = 0.56. Post-hoc comparisons showed that left responses were 8 ms faster when the stimulus appeared in the left hemispace than when it appeared in the right hemispace (339 vs. 347 ms, p = 0.05), while right responses were 16 ms faster when the stimulus appeared in the right hemispace than when it appeared in the left hemispace (320 vs. 336 ms, p = 0.002). Given the numerical difference in the size of two effects, we performed an independent sample t-test to assess whether they were statistically different. This analysis showed that the two effects did not differ, t(14)=-1.24 p = 0.23. t-tests showed that the two effects did not differ, *t*(14) = −1.24 *p* = 0.23. These results indicate that for both participants there was a significant Simon effect with respect to hemispace.

Experiment 1 assessed whether spatial coding occurs according to multiple frames of reference when individuals perform a joint Simon task. To this end, we manipulated stimulus position using a layout that provided multiple frames of reference for spatial coding^25^. Results showed that, for both Individual and Joint conditions, responses were faster for the most inner relative position of the stimulus than for the most outer relative position of the stimulus. This result confirms that when the stimulus set comprises a left and right element, left and right values are specified along both the egocentric and the relative dimensions^29^. However, differently from Roswarski and Proctor^25^, who reported faster reaction times for the most outer relative position, we found faster responses to the most inner relative position, indicating an effect of eccentricity (i.e. RTs increased as a function of retinal eccentricity along the horizontal meridian^30^). The discrepancy between our results and those reported by Roswarski and Proctor^25^ could be explained by the fact that in Roswarski and Proctor’s work participants performed an individual two-choice Simon tasks sitting in front of the screen, thus the eye and body midlines were aligned with the horizontal centre of the display. As consequence of this, for the outer locations all three spatial codes (i.e. hemispace, relative position and response position) were in agreement.

In the Individual condition, the Simon effect emerged neither with respect to hemispace nor with respect to relative stimulus position, as indicated by the lack of significant interactions involving the response position factor. For the Joint condition a significant Simon effect relative to hemispace emerged (i.e. left or right hemispace), suggesting that, when two individuals perform a joint Simon task, hemispace becomes the only relevant frame of reference.

### Experiment 2

Errors were 5.4% and 3.6% of the total trials for the Individual and Joint conditions, respectively, and were not further analysed. Trials slower than 3000 ms in the Joint condition were less than 1% and were excluded from the analyses. Since a preliminary analysis indicated that reaction times (RTs) were not normally distributed (Shapiro–Wilks test = 0.001), correct RTs were standardized by transformation into z-scores. A preliminary analysis on standardized RTs showed no main effect or interactions of participant’s position (i.e. left-seated vs. right-seated), all *p*s > 0.94, thus this factor was not included in the subsequent analyses.

Correct standardized RTs were submitted to a repeated-measures ANOVA with Hemispace (left vs. right), Relative stimulus position (left vs. right), and Response position (left vs. right) as within-participant factors, and Condition (Individual vs. Joint) as between-participants factor. The respective data are reported in Table 2. For sake of clarity, non-standardized RTs are reported. Following significant interactions, post-hoc comparisons were performed using the Newman-Keuls test.

The analysis revealed a main effect of Condition, *F*(1, 58) = 10.84, *p* = 0.002, *η*_*p*_^2^ = 0.16, with faster RTs in the Individual than in the Joint condition (481 vs. 535 ms, respectively). This result is probably due to the shorter response time allowed in the Individual condition as compared to the Joint condition. No other main effect was significant, all *p*s > 0.32. The interaction between Hemispace and Relative stimulus position was significant, *F*(1,58) = 19.53, *p* < 0.001, *η*_*p*_^2^ = 0.25. Post-hoc comparisons showed that, within each hemispace, responses to the most outer relative position of the stimulus (i.e. the left relative stimulus position in the left hemispace and the right relative stimulus position in the right hemispace; Fig. 1, panels A and D) were slower (512 and 513 ms for the left and the right hemispaces, respectively) than responses to the most inner relative position (i.e. the right relative stimulus position in the left hemispace and the left relative stimulus position in the right hemispace; Fig. 1, panels B and C; 501 and 506 ms for the left and the right hemispaces, respectively), all *p*s < 0.04. No difference was evident between the two inner positions and between the two outer positions (*p*s > 0.12).

The interaction between Hemispace and Response position, indicative of a Simon effect for hemispace, was significant, *F*(1,58) = 55.21, *p* < 0.001, *η*_*p*_^*2*^ = 0.49.Post-hoc comparisons showed that left responses were 24 ms faster when the stimulus appeared in the left hemispace than when it appeared in the right hemispace (497 vs. 521 ms, *p* < 0.001), while right responses were 19 ms faster when the stimulus appeared in the right hemispace than when it appeared in the left hemispace (497 vs. 516 ms, *p* < 0.001).

The interaction between Relative stimulus position and Response position, indicative of a Simon effect for the relative position, was significant, *F*(1, 58) = 6.14, *p* = 0.016, *η*_*p*_^*2*^ = 0.10. Post-hoc tests indicated that right responses were 7 ms faster when the stimulus appeared on the right than when it appeared on the left (503 vs. 510 ms, p = 0.05). The difference between the two stimulus positions did not reach significance for left responses (*p* = 0.50). No other interaction reached significance, all *p*s > 0.10.

Experiment 2 assessed whether the joint Simon Effect reported for the hemispace in Experiment 1 was due to the fact that co-actors provided a spatial reference point for coding the location of the response. To this end, participants performed a complementary task in which each actor executed a two-choice Simon task either alone or along with another participant. For both experimental conditions, results showed that responses were faster for the most inner relative position of the stimulus than for the most outer relative position. This result confirms that when the stimulus set comprises a left and right element, left and right values are specified along both egocentric and relative dimensions^25^,^29^. As in Experiment 1, these results indicate an effect of eccentricity^30^. Furthermore, for both conditions, the Simon effect occurred both with respect to the stimulus absolute position (i.e. left or right hemispace) and, for right responses only, with respect to its relative position within each hemispace (i.e. left or right relative position). Hence, when performing a complementary task in which each agent has two response keys at his/her disposal, spatial coding relies on multiple frames of reference, similarly to what occurs in a standard Simon task. The finding of a Simon effect with respect to relative location only for right responses is in line with previous studies reporting an advantage of the dominant hand when it operates in the corresponding space (e.g.^31^).

It should be noted that the entity of the Simon effect for the relative position found in our study (7 ms) is similar to the effect found in Roswarski and Proctor’s Experiment 3 (9 ms) when one single participant performed the two-choice task with red/green stimuli, as was the Simon effect with regard to hemispace (21 ms in our study vs. 29 ms in Roswarski and Proctor’s Experiment 3). Similarly to Roswarski and Proctor’s Experiment 3^25^, the effect emerging with respect to hemispace is larger than the effect emerging with respect to relative stimulus position. As suggested by Roswarski and Proctor^25^, multiple spatial codes are not formed automatically but they may depend on the specific encoding strategies used by participants which, in turn, may depend by the characteristics of the task context rendering one frame of reference more salient than the other.

## General Discussion

The present study was aimed at assessing whether, during complementary task performance, spatial coding is affected by the presence of another actor. Specifically, we investigated whether spatial coding in a joint version of the Simon task relies on multiple frames of reference, as occurs when a single individual performs the task. To this end, in two experiments the frame of reference for S-R spatial coding was systematically manipulated by presenting stimuli in four horizontal locations by using a display-control setting that allowed coding S-R correspondence with regard to multiple frames of reference.

The results of Experiments 1 clearly indicated that, when each participant has only one response at his/her disposal, spatial coding at the basis of the JSE does not rely on multiple frames of reference. Indeed, when paired participants performed a joint Simon task, stimulus position was coded according to its position in the hemispace (i.e. with reference to the centre of the screen) only. Moreover, results from Experiment 2 showed that, when co-agents could code response position according to their body midline, spatial coding relied on multiple frames of reference, similarly to when the Simon task is performed individually^25^, and no effect of the social context emerged.

Previous studies on multiple frames of reference for the individual Simon task^24^,^25^,^26^ reported that, when multiple frames of reference are present, stimulus location is coded with respect to the frame that is defined as more relevant. However, in all these studies hemispace was implicitly (i.e. by presenting a fixation cross or boxes only in the hemispace in which the stimulus occurred^24^,^25^) or explicitly (i.e. by instructing participants to respond to the stimulus based on its location with respect to hemispace; see^25^ Experiment 4) defined as the relevant frame. On the contrary, in our study no cues or instructions were given to define hemispace as the relevant frame of reference. Thus, it is possible that in joint tasks, hemispace is the relevant frame of reference according to which actions are coded. Further studies should investigate this issue. Moreover, the absence of multiple frames of reference in the joint Simon task used in Experiment 1 extends previous results showing that the representations guiding joint task performance might be quite different from the representations guiding individual performance (e.g.^12^,^32^,^33^).

Using an ecological display we demonstrated that, when the task was shared between two agents, knowledge about the position of the co-actor’s response, or the position of the co-actor himself/herself was represented and used as a reference for spatial response coding. In Experiment 2, since each agent had two response keys at his/her disposal and could hence code the response as right or left according to the body midline, even though the task was shared and the co-actor was physically present within the peri-personal space, the co-actor and his/her response did not represent a salient task-relevant event and did not serve as a reference for spatial coding.

Taking together the results of the present study are in line with the proposal that when the Simon task is shared, space may be used as an indication of whose turn it is^22^. Thus, the appearance of a stimulus on the left does not bring to the automatic activation of the left response, but rather is perceived as a stimulus signalling that the person sitting on the left is in charge of responding. As a consequence, participants are faster to respond to stimuli appearing in a corresponding position compared to those appearing in a noncorresponding position.

Notably it has been proposed^34^ that in joint action the spatial representations of both agents are re-calibrated, in analogy to the re-calibration of visual receptive fields due to tool use^35^. Thus, co-actors can perceive and act using a Shared Action Space (SAS^34^). Results of Experiment 1 speak in favour of this account. Indeed, results indicate that performing individual tasks within a social context allowed co-agents to represent the space of action as shared and thus, contrary to the individual context, to code stimuli and response positions with reference to the shared frame of reference (i.e. space).

The results of the present study may have important practical implications, especially considering the numerous real-life settings that may require different levels of complementarity between agents in performing specific tasks. Specifically, we believe they may provide useful insights on the influence played by the interaction between the social context (i.e. the presence of others) and task features on individual performance. If we consider the example taken from the aviation field reported in the Introduction, although speculative, our data might suggest that individual action-space is affected by the presence of an agent sitting in close proximity when pilot and co-pilot need to act in coordination in order to alternate themselves in performing the same task (i.e. flying the aircraft).

## Methods

### Participants

Thirty-two undergraduates (9 males; mean-age: 22.3 ± 2.2 years) from the University of Modena and Reggio Emilia took part in Experiment 1 and sixty undergraduates (19 males, mean-age = 20.1 ± 1.1 years) from the University of Bologna took part in Experiment 2. They received course credit for their participation. All were right-handed, reported normal or corrected-to normal vision and were naïve as to purpose of the experiment. The study was conducted in accordance with the ethical standards laid down in Declaration of Helsinki, and fulfilled the ethical standard procedure recommended by the Italian Association of Psychology (AIP). All procedures were approved by the Department of Communication and Economics of the University of Modena and Reggio Emilia and by the ethical commitee of the University of Bologna. Written consent was obtained from all participants and they were debriefed about the aim of the study at the end of the experiment.

### Apparatus, stimuli and display

The experiments were carried out in a dimly lit and noiseless room. Participants were seated facing a 17” LCD screen driven by a 700 MHz processor computer at a viewing distance of 70 cm. Stimulus presentation, response timing, and data collection were controlled by the E-Prime version 2 software (Psychology Software Tools, Inc).

**Figure 2:**
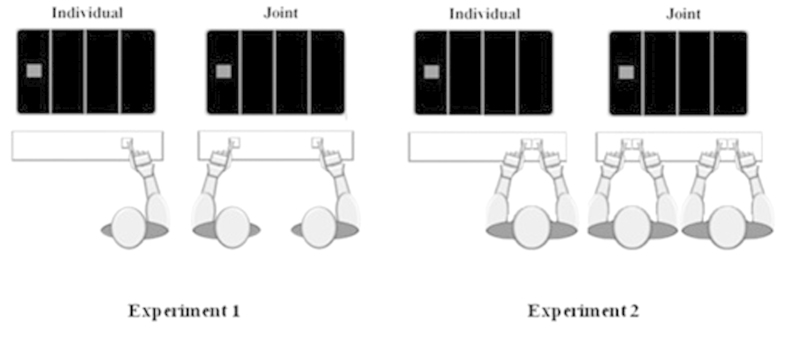
Schematic representation of the display and experimental conditions used in Experiment 1 (left panel) and Experiment 2 (right panel).

In both experiments, the display consisted of three white lines of 1 mm (0.09°of visual angle) in width and 300 mm (26.70° of visual angle) in height that were 116 mm (17.98°of visual angle) apart from one another. More specifically, one line was in the horizontal centre of the display, with a second line to the left of the central line and a third line to the right of the central line (Fig. 2). In Experiment 1, stimuli were red or green solid squares, while in Experiment 2 stimuli were red, green, yellow and blue solid squares (4.07° × 4.07°of visual angle). In both experiments, the stimulus was always presented in the centre between one of the outside lines and the centre line, or at an equal distance to the left of the leftmost line, or to the right of the rightmost line. Responses were executed by pressing the “z” or “-” keys of a standard Italian keyboard in Experiment 1 and the “z”, “x”,“-” or “.” keys in Experiment 2.

### Procedure

In both experiments, participants were randomly assigned to one of two experimental conditions (Individual vs. Joint). In the Individual condition of Experiment 1, each participant performed the task alone, sitting to the right or left from the centre of the screen. In the Joint condition of Experiment 1, participants were randomly paired and performed the task seating side-by-side, one to the left and one to the right of the centre of the screen (Fig. 2, left panel). For both conditions of Experiment 1 (i.e. Individual and Joint), the experimental procedure was as follows. First, the three lines appeared on the black display and served to demarcate the possible stimulus locations. Participants were required to fixate the line displayed in the centre of the display. After 1 s, the stimulus appeared in one of the four locations. The stimulus appeared with equal probability in each location. The lines and stimulus remained present until a response was made and for a maximum of 800 ms.The inter-trial-interval was 1 s, and it initiated immediately after the response was made. No feedback was provided. In both conditions, participants were required to respond to only one stimulus colour (red or green) by pressing the ipsi-lateral key (“z” or “-”) with the index finger of the outside hand. In the Individual condition, half of the participants were instructed to respond to the red stimulus, whereas the other half to the green stimulus. In the Joint condition, for half of the pairs, the participant on the right was instructed to respond to the green stimulus while the participant on the left was instructed to respond to the red one, while the other half experienced the opposite mapping (Fig. 2). Hence, in both conditions, participants performed a go/no-go Simon task. Instructions emphasized both the speed and the accuracy of the response.

In the Individual condition of Experiment 2, each participant was seated alone to the right or left of the centre of the monitor. In the Joint condition of Experiment 2, participants were randomly paired and seated side-by-side in front of the same monitor (Fig. 2, right panel). For both conditions, the lines appeared on the black display, remained on throughout the trial and served to demarcate the possible stimulus locations. Participants were required to fixate the line displayed in the centre of the display. After 1 s., a stimulus appeared in one of the four locations. The stimulus appeared with equal probability in each location. In the Individual condition, the lines and stimulus remained present until a response was made or for a maximum of 800 ms. In the Joint condition, the lines and the stimulus remained present until a response was made. The inter-trial-interval was 1 s, and it was initiated immediately after the response was made. For both conditions, participants were instructed to respond to only two of the stimulus colours (red and green or yellow and blue) by pressing the keys at their disposal with the index fingers of the right and left hand. Participants were randomly assigned to the left or right seat. Participants seated on the right were instructed to respond to the red and green stimuli by pressing either the “z” or the “x” keys, whereas the participants seated on the left were instructed to respond to the yellow and blue stimuli by pressing either “-” or the “.” keys. For both left- and right-seated participants the stimulus-response mapping was counterbalanced across participants. Instructions emphasized both the speed and the accuracy of the response.

For both experiments, the experimental session consisted of 384 experimental trials that were divided into four blocks of 96 trials each and a practice session of 24 trials was given at the beginning of the experiment. Each experiment lasted about 25 minutes.

## Additional Information

**How to cite this article**: Ciardo, F. *et al*. Action-space coding in social contexts. *Sci. Rep.* **6**, 22673; doi: 10.1038/srep22673 (2016).

## Figures and Tables

**Table 1:** Experiment 1: Mean correct reaction times (and standard error) in ms as a function of Condition (Individual vs. Joint), Hemispace (left vs. right), Relative stimulus position (left vs. right) and Response position (left vs. right).

**Individual**			**Joint**		
	**Hemispace**			**Hemispace**	
	**Left**	**Right**		**Left**	**Right**
Left Relative position			Left Relative position		
Left response	364 *(16)*	358 *(16)*	Left response	348 *(16)*	338 *(16)*
Right response	347 *(16)*	334 *(16)*	Right response	342 *(16)*	315 *(16)*
Right Relative position			Right Relative position		
Left response	364 *(17)*	365 *(17)*	Left response	329 *(17)*	357 *(17)*
Right response	336 *(17)*	344 *(17)*	Right response	330 *(17)*	324 *(17)*

**Table 2:** Experiment 2: Mean correct reaction times (and standard error) in ms as a function of Condition (individual vs. joint), Hemispace (left vs. right), Relative stimulus position (left vs. right) and Response position (left vs. right).

**Individual**			**Joint**		
	**Hemispace**			**Hemispace**	
	**Left**	**Right**		**Left**	**Right**
Left Relative position			Left Relative position		
Left response	473 *(13)*	489 *(13)*	Left response	523 *(13)*	545 *(13)*
Right response	503 *(12)*	462 *(16)*	Right response	548 *(12)*	527 *(16)*
Right Relative position			Right Relative position		
Left response	469 *(11)*	499 *(12)*	Left response	525 *(11)*	552 *(12)*
Right response	479 *(11)*	472 *(14)*	Right response	533 *(11)*	529 *(14)*
